# Intracellular Sources of ROS/H_2_O_2_ in Health and Neurodegeneration: Spotlight on Endoplasmic Reticulum

**DOI:** 10.3390/cells10020233

**Published:** 2021-01-25

**Authors:** Tasuku Konno, Eduardo Pinho Melo, Joseph E. Chambers, Edward Avezov

**Affiliations:** 1 Department of Clinical Neurosciences, UK Dementia Research Institute, University of Cambridge, Cambridge CB2 0AH, UK; 2 CCMAR—Centro de Ciências do Mar, Campus de Gambelas, Universidade do Algarve, 8005-139 Faro, Portugal; emelo@ualg.pt; 3 Cambridge Institute for Medical Research, University of Cambridge, Cambridge CB2 0XY, UK; jec202@cam.ac.uk

**Keywords:** reactive oxygen species, hydrogen peroxide, endoplasmic reticulum, redox signalling, antioxidants, neurodegeneration, dementia

## Abstract

Reactive oxygen species (ROS) are produced continuously throughout the cell as products of various redox reactions. Yet these products function as important signal messengers, acting through oxidation of specific target factors. Whilst excess ROS production has the potential to induce oxidative stress, physiological roles of ROS are supported by a spatiotemporal equilibrium between ROS producers and scavengers such as antioxidative enzymes. In the endoplasmic reticulum (ER), hydrogen peroxide (H_2_O_2_), a non-radical ROS, is produced through the process of oxidative folding. Utilisation and dysregulation of H_2_O_2,_ in particular that generated in the ER, affects not only cellular homeostasis but also the longevity of organisms. ROS dysregulation has been implicated in various pathologies including dementia and other neurodegenerative diseases, sanctioning a field of research that strives to better understand cell-intrinsic ROS production. Here we review the organelle-specific ROS-generating and consuming pathways, providing evidence that the ER is a major contributing source of potentially pathologic ROS.

## 1. Introduction

Substantial quantities of reactive oxygen species (ROS), H_2_O_2_ in particular, are produced intracellularly. H_2_O_2_ is generated endogenously as a functional entity and as a by-product of reducing/oxidising (redox) chemistry: H_2_O_2_ can be generated by regulated enzymatic processes to serve as a signal molecule, fuel protein disulfide bonding or as a controlled toxin in immunological-activity. Imperfections in electrochemical reactions can result in unintended H_2_O_2_/ROS (e.g., mitochondrial respiration chain with oxygen as terminal electron acceptor). Some ROS, specifically free radicals, are short-lived as a product of their high reactivity with biological macromolecules, including nucleic acids and lipids, which in turn can render them hazardous. By contrast, H_2_O_2_, a non-radical ROS, is less reactive with biomolecules and as such has a relatively long biological lifespan (cellular half-life of ~1 ms) [1,2]. Yet H_2_O_2_ is also capable of producing harmful radicals, through fenton chemistry [3]. Therefore, ROS generating pathways have co-evolved with mechanisms to control their spatio–temporal distribution. Thus, cellular compartments handling the protein-encoding nucleic acids maintain a strictly reducing environment (redox potential estimated at −300 mV and −280 mV in the nucleus and cytoplasm, respectively [4]) (see glossary). These compartments are equipped with ROS quenching enzymes, e.g., catalase and glutathione peroxidases. The reducing nature of such environments allows transient and localised usage of H_2_O_2_ as a second messenger. As such, H_2_O_2_ complements the cell’s repertoire of second messengers as it can induce rapid and reversible functional changes to proteins through intra/inter molecular disulfide bonding and other cysteine modifications such as sulfenylation and sulfonation. This principle is exemplified by a bacterial H_2_O_2_ sensing system, where the key protein, OxyR contains a peroxidatic cysteine residue, surrounded by positive charges, that is preferentially reactive with H_2_O_2_. This reaction results in a sulfenic intermediate that is highly reactive with the thiolate of a second “resolving” cysteine, producing an intramolecular disulfide that converts the protein into a functional transcription factor that activates the production of ROS-antagonising enzymes [5]. The kinetic advantage of H_2_O_2_, in such a system, over the reducing forces of the cell allows this reaction to prevail even when its concentration is 2–3 orders of magnitudes lower than the antagonising reductants [6]. As such, OxyR has been utilised as a template in engineering a genetically encoded fluorescent H_2_O_2_ probe, HyPer, sensitive to physiological H_2_O_2_ concentrations [7].

The short-lived nature of H_2_O_2_ relative to the biomolecules it reacts with and the overwhelming cellular reductive force ensure the locality and transience of H_2_O_2_-mediated signalling events. Several signalling pathways of mammalian cells take advantage of this chemistry, enzymatically producing local H_2_O_2_ that can activate factors such as kinases. For instance, oxidation of cysteine residues in SRC, a proto-oncogene non-receptor tyrosine kinase, activates this enzyme, regulating cell proliferation and survival by activating downstream pathways such as ERK and AKT signalling [8]. Several transcription factors, such as NF-κB and KEAP1-NRF2, also sense H_2_O_2_ to activate transcriptional programs [9,10].

The reducing, ROS disfavouring environment of the nucleus, cytoplasm and mitochondria contrasts with a relatively oxidising milieu in the organelles of the secretory pathway. The endoplasmic reticulum (ER) lumen, in particular, maintains an oxidising environment similar to that of the extracellular conditions. This is favoured by the relatively oxidising reduction potential of glutathione (*E_GSH/GSSG_*), a measure of redox conditions, estimated at −208 mV in the ER [11,12]. Moreover, the ER maintains a relatively high turn-over of H_2_O_2_ as its oxidative protein folding machinery both produces and utilises H_2_O_2_ continuously. H_2_O_2_ apparent confinement to the ER (discussed herein) can be compromised when redox homeostasis is perturbed [13,14].

This notion places the ER in the spotlight as a potential source of ROS associated with pathologies, including those associated with neurodegenerative diseases. Excessive ROS load entails damage of structural and functional macromolecules including lipids, protein, RNA, and perhaps with the most severe repercussions to DNA, where a single unrepaired damage event can impact on all its downstream products. As such, compromised insulation of organelles that maintain naturally high ROS (such as ER) may contribute to pathological elevations in ROS.

With an emphasis on the ER, here we review the findings contributing to the current understanding of organelles’ ROS producing/consuming pathways and discuss how an imbalance in the activity of ROS generating and antagonising pathways as well as organellar ROS permeability dysregulation can lead to an increase in their steady-state load. The intracellular sources of H_2_O_2_ are discussed in the context of their association with neurodegenerative diseases.

## 2. ER Sources of ROS/H_2_O_2_

The ER is devoid of ROS-vulnerable genetic material and therefore, predictably, can tolerate a high abundance of ROS. In addition, H_2_O_2_ reactivity with most protein functional groups is energetically unfavourable, suggesting it is unlikely to significantly contribute to protein misfolding directly [15]. ER oxidative protein folding both generates, and is at least partially fuelled by, H_2_O_2_ (described in detail below) and basal ER ROS content is reported to be relatively high [13]. This is unsurprising, as approximately 30% of cellular proteins enter the ER, many of them en route to the cell membrane or secretion. A 1970s ex vivo study using rat liver homogenate reports that H_2_O_2_ in the microsomal fraction, which contains the ER predominantly, accounts for 45% of the cell total, three times that derived from mitochondrial extracts of the same samples [16,17]. Thus, the ER appears uniquely capable of producing and utilising H_2_O_2_ as fuel for enzymatic oxidative folding process on a massive scale.

**Figure 1:**
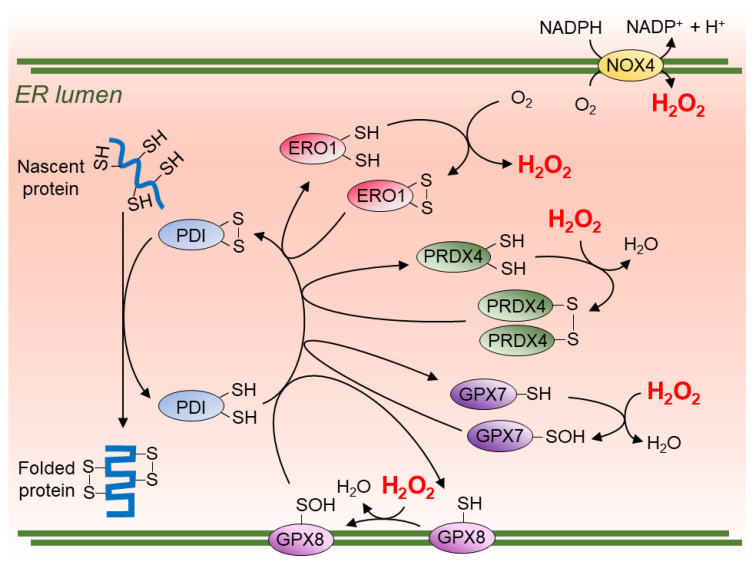
Schema of pathways producing and utilising endoplasmic reticulum (ER)-derived H_2_O_2_. Reduced thiols (-SH) of cysteine residues in nascent proteins are oxidised by catalytically competent protein disulphide isomerase (PDI)s to promote protein folding with native disulphide bonds (S-S). PDI can be re-oxidised by ERO1, with flavin adenine dinucleotide (FAD) embedded in its active site, which reduces molecular oxygen (O_2_) to hydrogen peroxide (H_2_O_2_). ER-resident antioxidants PRDX4, GPX7, and GPX8 oxidise their catalytic domain by utilising H_2_O_2_ (-SOH = sulfenic acid) and mediate “the disulphide relay” to PDI. NOX4 produces H_2_O_2_ over superoxide on the ER membrane, unlike other NOX family proteins.

The core of the ER oxidative folding machinery is a group of oxidoreductase-chaperone enzymes of the protein disulphide isomerase (PDI) family, which functionally interact with their substrates via redox-active CXXC motifs of their catalytic thioredoxin domains (Figure 1) [18]. PDI itself catalyses disulphide bond formation in substrate proteins, leading to reduction of PDI CXXC motifs that are in turn reoxidised by ER oxidoreductin 1 (ERO1). The later produces H_2_O_2_ as two electrons per thiol pair flow from PDIs to molecular oxygen [19]. In this process, ERO1 is estimated to consume 25% of the oxygen available in the cell [20,21,22]. Yet the by-product of this reaction, H_2_O_2_, can be consumed by another process that drives PDI reoxidation, catalysed by the ER-localised peroxiredoxin PRDX4 (pathway described below). Notably, even though ERO1 gene expression is up-regulated in response to stimuli such as hypoxia and the unfolded proteins response (UPR) [23,24,25,26], knockdown of ERO1 gene leads to a resistance to tunicamycin-induced ER stress in worms and ER stress-induced apoptosis in mouse macrophages [27,28]. It seems that up-regulation of ERO1 results in excessive ROS production rather than improving oxidative folding capacity. Indeed, excess of ERO1 activity caused by hyperactive mutants or overexpression has been reported to increase ROS production and cell death [29,30]. Noteworthily, knockdown of ERO1 in worms extends their lifespan by 32% and is accompanied by a reduction in peroxide levels [31]. Thus, through its role in oxidative protein folding, excessive ERO1 activity contributes to an ER-emanating ROS hazard.

Although ERO1 deficit is lethal in yeasts [32,33], this gene is at least partially dispensable in mammals (knockout of all ERO1 isoforms, *Ero1a* and *Ero1b*, gives rise to only a mild phenotypic defect in mice [34,35]). In the absence of ERO1, the oxidative folding process can be sustained by peroxiredoxin 4 (PRDX4), an ER-localised antioxidative enzyme that catalyses PDI reoxidation [36]. PRDX4 scavenges H_2_O_2_, forming water with the concomitant relay of oxidative equivalent to PDIs (Figure 1). In normal conditions, PRDX4 works presumably in parallel to ERO1 in oxidative recycling of PDI. Fuelled by H_2_O_2_ generated as a by-product of ERO1 activity, PRDX4 seems to work alongside ERO1 as a wasteless system of transferring electrons from cysteine thiols to oxygen generating water in the process. The robustness of ER oxidative protein folding in higher organisms may be in part attributed to the evolutionary acquisition of these parallel pathways (the PDI-PRDX4 pathway is absent in yeast [36]). As such, ER-accumulation of H_2_O_2_ that could have detrimental effects in other cellular compartments is dealt with “in house” by the ER, through a mechanism that further promotes oxidative protein folding capacity.

The triple knockout mouse model that lacks *Prdx4*, *Ero1a* and *Ero1b* is viable, whilst showing a compromised oxidative folding kinetics [6] and a scurvy-like phenotype, presumably due to impaired collagen maturation [37]. This provides evidence to support that additional proteins are capable of driving oxidative protein folding in the ER (Figure 1).

The importance of PRDX4-mediated H_2_O_2_ quenching in the ER is highlighted by phenotypic consequences of its genetic manipulation: Its overexpression led to protection against glutamate-induced neuronal cell death, increased insulin production, and inhibition of adipogenesis [38,39,40]. *Prdx4* knockout mice showed phenotypes such as abnormal spermatogenesis and susceptibility to drug-induced colitis, indicating a systemic requirement for this protein [41,42]. Moreover, the expression level of PRDX4 positively correlates with the prognosis of patients with lung adenocarcinoma [43]. The apparent ability of cells to maintain ER oxidative protein folding in the face of PRDX4 loss suggests that ERO1 activity is likely sufficient to perform this function, albeit driving further H_2_O_2_ production.

Besides PRDX4, ER H_2_O_2_ is also utilised by ER-resident glutathione peroxidases, GPX7 and GPX8 (ER luminal and lumen-facing membrane anchored, respectively [44]). Both can oxidise PDI in the presence of H_2_O_2_ and interact with ERO1, catalysing oxidative protein folding [29,44,45]. They can also protect insulin-secreting cells from ER stress induced by saturated fatty acids (FFAs) [45]. Metformin, a therapy for diabetes, is reported to provide antiaging and cognitive decline-diminishing benefits that are thought to be mediated by the upregulation of GPX7 [46]. Maintaining GPX7 levels seems to be important to protect from hyperactivation of ERO1 and ER stress triggered by homocysteine, which causes hyperhomocysteinemia-related vascular diseases [47]. *Gpx7* knockout mice show systemic oxidative stress, carcinogenesis and shortened lifespan [48]. Given that GPX7 can protect both the ER and nuclear DNA from oxidative damage, the effects of ER ROS appear to reach beyond the lumen of the organelle [49]. Supporting this view, removal of GPX8 increases H_2_O_2_ levels in the cytosol, indicating that GPX8 may play a role in preventing leakage of H_2_O_2_ from the ER [14].

The membrane localised enzyme NADPH oxidase 4 (NOX4) provides another source of ER H_2_O_2_. This enzyme preferentially produces H_2_O_2_ rather than superoxide through a highly conserved histidine residue in its extracytosolic loop (E-loop), not present in other NOX family proteins [50]. NOX4-derived H_2_O_2_ drives multiple biological events inside and outside the ER (e.g., Ras signalling activation through ER Ca^2+^ efflux by sarco/endoplasmic reticulum calcium-ATPase (SERCA) oxidation [51,52], H_2_O_2_ traversal of ER membrane in this extra-ER activity is likely mediated by aquaporins, see discussion in the next section). Although NOX4 is found in other organelles, this protein has been shown to be predominantly expressed on the ER membrane in human neuroblastoma SH-SY5Y cells, human hepatocyte Hep G2 cells, and human umbilical vein endothelial (HUVEC) cells [51,53]. NOX4 interacts with calnexin, an ER membrane lectin-chaperone, with a degree of dependence on this factor: elimination of calnexin reduces NOX4 expression level as well as ROS production [54]. Moreover, NOX4 also interacts with PDI [55], and its interaction controls uncoupling of endothelial nitric oxide synthase (eNOS) and expression of β-galactosidase, which are involved in ageing of vascular endothelial cells [56].

Protective roles have been suggested for ER-localised NOX4 activity. NOX4 has been suggested to drive prolonged integrated stress response signalling by oxidatively inhibiting protein phosphatase 1 (PP1) and protecting against acute kidney injury in vivo [57]. 

The lumen of the ER appears to be endowed with an additional crucial layer of controlling H_2_O_2_ load. Measurements using an ER-tuned genetically encoded H_2_O_2_ sensor pointed to the role of reduced glutathione in buffering the load of ER-intrinsically generated H_2_O_2_ [13]. Relatively high ER-glutathione (approximately 10–15 mM total, mostly reduced [58,59]) may compensate for poor kinetics of GSH–H_2_O_2_ reactivity. Regulation of ER redox by glutathione seems to be mediated by its flux and Ca^2+^ mobilisation, at least in part [60]. However, H_2_O_2_-degrading enzymes, such as the ER-resident PRDX4 and glutathione peroxidases GPX7 and GPX8, are significantly more efficient hydrogen peroxide-degrading catalysts than GSH alone. However, above a threshold concentration, calculated to be within the range of concentrations at which H_2_O_2_ acts as a second messenger, chemical reduction of H_2_O_2_ by GSH becomes kinetically competitive with enzymatic activity, aiding the maintenance of H_2_O_2_ levels. Accordingly, when glutathione is depleted, levels of H_2_O_2_ in the ER augment, and the viability of cells with reduced cytosolic antioxidant capacity (e.g., a pancreatic β-cell model, with natural catalase deficiency) becomes strongly compromised, particularly when H_2_O_2_ production is further stimulated through the biosynthesis of disulphide-containing substrates (e.g., pro-insulin) [13]. This highlights the physiological relevance of the chemical reduction of H_2_O_2_ by GSH and further suggests that the ER constitutes a significant source of pathological ROS.

### H_2_O_2_ Transport Across the ER Membrane

The abundance of H_2_O_2_ in the ER suggests that the ER membrane likely limits its conductance towards other, potentially more ROS-vulnerable cellular compartments. The H_2_O_2_ quenching mechanisms on the cytosolic side of the ER membrane likely contribute to the insulation. However, mechanistic details of what promotes or limits H_2_O_2_ permeability over membranes remains only partially understood. A view point in favour of unlimited H_2_O_2_ transit across the ER membrane was expressed [61]. 

Since artificially imposed H_2_O_2_ removal in other organelles does not affect H_2_O_2_ content of the ER, the influx of H_2_O_2_ into the ER from other organelles seems to be tightly restricted at steady state [6]. However, in light of measurements showing that ER-derived H_2_O_2_ can leak to the cytosol under conditions of luminal H_2_O_2_ overload, potentially causing oxidative damage in other organelles [13,14], transport of ER H_2_O_2_ across the membrane is a plausible toxicity-determining parameter. The general principles of controlled material exchange between organelles through passive channels or active transporters seem to apply in the case of H_2_O_2_: aquaporins (AQPs, described initially as water channels) have been suggested to conduct H_2_O_2_ across membranes [62,63,64,65].

In mammals, the AQP family of proteins consists of thirteen isoforms. They share a common topology of six transmembrane helices, five loops (three extracellular and two intracellular), and two highly conserved asparagine–proline–alanine (NPA) motifs. Assembled as tetramers, these proteins form an hourglass-like functional pore [66]. They are classified by three sub-groups; classical AQPs (AQP0, AQP1, AQP2, AQP4, AQP5, AQP6, AQP8), aquaglyceroporins (AQP3, AQP7, AQP9, AQP10), and unorthodox or superaquaporins (AQP11, AQP12), based on their substrates. In addition, recently a new category—peroxiporins has been identified (AQP1, AQP3, AQP5, AQP8, AQP9, AQP11), whose common feature is the ability to transport H_2_O_2_ with a degree of preference [63,67,68]. It is worth noting that all AQPs can potentially enable transport of H_2_O_2_ to some extent [69]. 

As all AQPs start their journey through the secretory pathway at the ER, each can potentially contribute to H_2_O_2_ transport across its membrane. Notably, AQP8 was explicitly shown to mediate H_2_O_2_ transport across the ER membrane [67]. Partial ER-localisation of AQP4 and AQP9 have also been observed [68]. Further, human AQP11 showed ER localisation and the capacity to transport H_2_O_2_ across the ER membrane [70,71].

The precise function of peroxiporins on the ER membrane currently remains obscure. One can speculate that AQPs may maintain redox homeostasis or even support cellular signalling by modulating H_2_O_2_ efflux from the ER, as well as the influx to the ER. This is indirectly supported by the finding that the expression level of NOX2 and renal oxidative damage are augmented in *Aqp11* knockout mice [72]. Recent findings on hydrogen sulphide (H_2_S)-mediated regulation of an ER aquaporin’s conductivity support such a possibility [73].

It is conceivable that regulated and localised H_2_O_2_ conductivity of the ER membrane may constitute a mechanism for time-space specific releases of H_2_O_2_ from its “ER-store”. Such a mechanism, hitherto uncharacterized, could be considered analogous to regulated calcium releases from ER stores. In the case of the short-lived H_2_O_2_ the “storage” is afforded by its constant high productions rate in the confined space of the ER. 

## 3. Non-ER Sources of ROS/H_2_O_2_

### 3.1. Mitochondrial ROS/H_2_O_2_

Though the mitochondrial matrix maintains a relatively reducing environment (redox potential of −300 mV [4]), it is thought to constitute a major source of intracellular ROS, as a by-product of its multicomponent electrochemistry machinery. However, only 15% of total H_2_O_2_ was accounted for in a mitochondrial fraction of cell extracts [16,17]. This is consistent with the abundant and diverse antioxidant enzymology of the organelle, affording protection to other organelles and its precious DNA content from oxidative damage. 

Mitochondria synthesise ATP through oxidative phosphorylation, coupled to the electron transport chain (ETC). Leakage of electrons from the ETC, as well as other related enzymes, leads to a partial reduction of oxygen to form superoxide, and it is subsequently converted to H_2_O_2_ (a less reactive oxidant) and oxygen by superoxide dismutase 1 (SOD1) in mitochondrial intermembrane space, and SOD2 in mitochondrial matrix. Although most of the oxygen in the cell is consumed by mitochondria, only 1–2% seems to be converted to ROS [17]. Systemic homozygous knockout of SOD2 in mice led to pre-weaning lethality accompanied by multiple tissue dysfunctions such as cardiomyopathy, fatty liver, metabolic acidosis, and neurodegeneration [74,75]. In contrast, overexpression of both SOD2 and catalase protects the cell from antiretroviral-induced oxidative stress and cardiomyopathy [76].

To date, eleven ETC-related enzymes have been identified as the source of ROS in mitochondria [77,78]. Four of them are present in distinct 2-oxoacid dehydrogenase complexes, which catalyse the oxidative decarboxylation of different 2-oxoacids to the corresponding acyl-CoA and NADH. These are 2-oxoglutarate dehydrogenase complex (OGDHC), pyruvate dehydrogenase complex (PDHC), branched-chain 2-oxoacids dehydrogenase complex (BCOADHC), and 2-oxoadipate dehydrogenase complex (OADHC). ROS production from these complexes seems to be much higher than that from complex I, which has been previously considered as one of the major mitochondrial ROS sources [79]. Complex I (NADH ubiquinone oxidoreductase) generates H_2_O_2_ at a flavin-containing site and a quinone-binding site. Notably, an improved genetically encoded H_2_O_2_-specific sensor (HyPer7), detected complex1-derived H_2_O_2_, which is driven by its selective inhibitor rotenone, appears to be released toward the mitochondrial matrix side [80]. The remaining four of the ROS sources are located in dehydrogenases, linked to ubiquinone: dihydroorotate dehydrogenase (DHODH), the electron-transferring flavoprotein (ETF)–ubiquinone oxidoreductase system (ETF-QO), mitochondrial glycerol-3-phosphate dehydrogenase (mGPDH) as well as complex II (succinate dehydrogenase, SDH). ROS can also emanate from the outer ubiquinone-binding site of complex III [81].

Furthermore, mitochondria-localising NADPH oxidase can provide a source of H_2_O_2_. NOX4 is predominantly localised to mitochondria in certain cells such as mouse podocytes, mesangial cells and kidney cortex [82,83]. Mitochondrial NOX4-derived H_2_O_2_ was implicated in ageing-dependent aortic stiffening and drug resistance [84,85]. 

Of interest, cardiomyopathy triggered by the ablation of desmin, a major muscle-specific intermediate filament protein, is ameliorated by catalase overexpression but deteriorated by SOD2 overexpression [86]. This can be explained by either augmenting mitochondrial H_2_O_2_ or diminished superoxide and implies that balancing mitochondrial ROS maintains cellular homeostasis. Indeed, mitochondrial ROS seems to regulate hypoxic signalling [87]. In addition, mitochondrial ROS are reported to be required for expressions of hypoxia-inducible genes such as erythropoietin and Hypoxia-inducible factor 1 (HIF-1) [88], as well as enhancing cytosolic calcium levels in pulmonary arterial smooth muscle cells (PASMCs) during hypoxic pulmonary vasoconstriction (HPV) [89,90].

Notably, the majority of proteins in mitochondrial intermembrane space are known to contain disulfide bonds as a post-translational modification [91], and an electron relay mediated by ERV1, a mitochondrial FAD-linked sulfhydryl oxidase which mediates disulfide bond formation, results in H_2_O_2_ production analogous to the ERO1-pathway. H_2_O_2_ produced by ERV1 is scavenged by cytochrome C, likely forming another mode by which mitochondria are protected from the toxicity of H_2_O_2_ [92]. Uncoupling proteins (UCPs), the modulators of thermogenesis by uncoupling mitochondrial proton gradient and ATP synthesis, also seem to be important in mitochondrial H_2_O_2_ production regulation [93,94,95].

### 3.2. Peroxisomal ROS/H_2_O_2_

Peroxisomes are single-membrane-bounded organelles present in most eukaryotes. A variety of oxidation reactions, such as β-oxidation of fatty acids, biosynthesis of cholesterol, and metabolism of amino acids and purines are carried out in this organelle. Accordingly, the production of peroxisomal ROS is reportedly mediated by: xanthine oxidase, D-amino oxidase, urate oxidase, and Acyl-CoA oxidase [96]. Despite its relatively small size, the peroxisome is estimated to generate about 35% of total intracellular H_2_O_2_ and therefore is considered as a major source of intracellular ROS [97,98,99]. 

Peroxisomal ROS can function as a signal molecule and seem to be functionally associated with autophagy in particular. For instance, the negative regulators of mammalian target of rapamycin complex 1 (mTORC1), tuberous sclerosis complex 1 (TSC1) and 2 are localised to peroxisomes, where they suppress mTORC1 signalling and thus induce autophagy in response to ROS [100]. Moreover, peroxisomal ROS induce selective autophagy of peroxisomes themselves (pexophagy) [101]. Ataxia-telangiectasia mutated (ATM), a serine/threonine kinase responsive to DNA damage, is also activated at peroxisomes in response to peroxisomal ROS to induce autophagy and pexophagy [102]. It also has been suggested that peroxisomal ROS serve as a measure for quality control of the organelle’s function [103]. 

Use of a peroxisome-targeted H_2_O_2_ sensor revealed that peroxisomal redox milieu is kept reductive at steady-state despite abundant ROS productions [104]. This can be attributed to abundant peroxisome-resident antioxidative enzymes such as catalase, SOD1, SOD2, PRDX1, PRDX5, epoxide hydrolase, a peroxisomal membrane protein 20 (PMP 20), and potentially a GPX [105]. Although no subtype of GPX has been identified in mammals as of yet, Gpx1 has been found in the peroxisomal matrix in yeast [106]. Indeed, the neuronal-specific ablation of peroxin 5 (*Pex5*, a peroxisomal biogenesis factor) causes peroxisome dysfunction and axonal myelin loss without causing oxidative stress in the central nervous system [107]. Furthermore, deficiency of PEX5 and PEX12 shifts the localisation of catalase from peroxisome to cytoplasm, and protects cells from oxidative damage by preferentially removing cytosolic ROS [108]. Thus, although peroxisomes are a significant source of ROS, they do not seem to be a critical source of oxidative stress, unless their multiple ROS quenching enzymes fail collectively.

### 3.3. Nuclear ROS/H_2_O_2_

The nucleus is the most reducing organelle in the cell [109], a fact not incongruous with its function of storing DNA, a most precious and oxidation sensitive cellular component. The reductive nuclear milieu is thought to be fuelled by thiol reductants such as thioredoxin-1 (TRX1), GPXs, PRDXs, glutathione (GSH) and specific isoforms of glutathione S-transferases. These reductants seem to scavenge nuclear ROS and function in the nucleus as well as other subcellular localisations. For instance, TRX1 localises to both nucleus and cytosol at steady state. However, in the presence of excess ROS, TRX1 preferentially localises in the nucleus [110]. Redox potential shift of nuclear TRX1 (about 60 mV oxidation from −300 mV at steady state) indicates that TRX1 attends to the oxidative stress in the nucleus [110]. Moreover, cytosolic TRX1 can interact with apoptosis-inducing factor (AIF) by forming a disulphide bond that facilitates delivery of AIF to the nucleus [111]. After this delocalisation, the reduction of TRX1–AIF complex releases AIF in the nucleus where it functions to promote apoptosis. 

GSH accumulates in the nucleus during cell proliferation and spreads throughout the cell when cells are confluent. [112,113]. It can be speculated that this serves as a defensive measure to protect the nucleus from oxidative stress, as intracellular ROS increase during mitosis [114]. Indeed, depletion of GSH impairs cell proliferation [115]. GSH not only directly removes nuclear ROS but also introduces glutathionylation to nuclear proteins, such as histone H3, to regulate nucleosome stability by blocking the irreversible oxidation of cysteine residues [116]. The level of glutathionylation of H3 is high in rapidly proliferating cells such as cancer cells and low in aged cells, suggesting this redox event may be relevant to ageing and diseases.

Despite the abundance of nuclear reductants, ROS can unavoidably oxidise DNA in the nucleus. 8-Oxo-2’-deoxyguanosine (8-oxo-dG) is the most common product of DNA oxidation and has long been considered a useful, if somewhat biologically uninteresting, marker of oxidative damage. However, studies using genetic ablation of 8-oxoguanine DNA glycosylase 1 (OGG1), an enzyme that binds and removes 8-oxo-dG, have demonstrated a direct relevance of 8-oxo-dG to physical dysfunctions such as age-associated neuronal loss and susceptibility to a dopaminergic toxin 1-methyl-4-phenyl-1,2,3,6-tetrahydropyridine (MPTP) [117,118]. Such phenotypes are likely mediated by DNA methylation status because 8-oxo-dG recruits a DNA demethylase ten-eleven translocation 1 (TET1) via OGG1 [119]. Indeed, a negative correlation between 8-oxo-dG and 5-methyl cytosine (5mC), a methylated cytosine involved in gene transcriptions and various disorders, has been reported in gliomas [120]. Moreover, some gene expressions are upregulated by 8-oxo-dG on their loci promoter [121,122,123,124,125,126,127].

NOX4 appears as a significant nucleus-intrinsic source of ROS. Nuclear ROS emanating from NOX4 have been demonstrated to cause DNA damage, relevant to diseases such as myelodysplastic syndromes and viral infection [128,129], with a conceivable extension to neurodegeneration-associated DNA damage. Knockdown of *NOX4* in human endothelial cells diminishes DNA damage and expanded cellular lifespan, displaying a strong relevance to senescence [130]. Intriguingly, NOX4 activity is negatively regulated by FYN, a tyrosine kinase belonging to SRC family kinases, implying programmed regulation of nuclear ROS by NOX4 as a result of cellular signalling, which seems to be relevant to cardiac remodelling [131].

### 3.4. Golgi ROS/H_2_O_2_

There are fewer reports related to ROS production and oxidative stress in the Golgi apparatus than perhaps any other organelle. Since the main functions of the Golgi are secretory proteins’ glycosylation and sorting, studies exploring redox reactions in this compartment are relatively infrequent. However, the Golgi’s oxidative environment, which is equivalent to that of the ER, supports the plausibility of ROS production in this compartment [132].

Quiescin-sulfhydryl oxidases (QSOXs), flavin-linked sulfhydryl oxidases, are the most likely candidate for Golgi ROS production. All QSOX isoforms possess an ER signal peptide and are transported from the ER to the Golgi like generic secretory proteins lacking ER retention signals. Immunohistochemical analysis has also indicated a significant Golgi localised pool of this protein [133,134,135,136]. QSOX isoforms consist of two thioredoxin domains containing CxxC motifs and FAD-binding domain at the N-terminus. As such, they can oxidise thiol groups to form disulphide bonds with the reduction of oxygen and production of H_2_O_2_, much like PDI and ERO1 in the ER [137]. Indeed, a recent report demonstrates QSOX1-dependent H_2_O_2_ production in human placenta-derived trophoblast and involvement of this protein in the pathogenesis of preeclampsia [138]. On the other hand, QSOX1 overexpression protects rat pheochromocytoma PC12 cells from oxidative stress-induced cell death, although it has also been reported to slow down cellular proliferation [139], hence potential benefits of QSOXs (and Golgi ROS in general) may depend on tissue and cell type. Moreover, upregulation of QSOXs expression is found in several types of cancers such as breast, pancreas, and prostate cancers, suggesting the relevance of this gene to cancer development [140,141]. Interestingly, higher expression of QSOX1 seems to reduce tumorigenesis and is associated with better outcomes for breast cancer patients (relations to known functions of QSOXs remains unclear) [142]. Exogenously expressed QSOX1 restores impaired viability and disulphide bond formation capacity in Ero1p deficient yeast, suggesting that QSOX can rectify oxidative folding defects in proteins that, notwithstanding the ER quality control system, make it to the Golgi [136]. On the other hand, QSOX1 knocked-down fibroblasts fail to incorporate an extracellular matrix (ECM) protein—laminin, but not collagen IV and fibronectin [133]. Markedly, this defect is rescued by the addition of recombinant QSOX1 to the cell culture media [133]. These suggest that QSOX1, and its single-chain arrangement of ERO1 and PDI equivalents may specialise on a subset of secretory proteins and act extracellularly.

The existence of other redox enzymes further implies the importance of redox regulation in the Golgi. In yeast, glutaredoxin 6 and 7 (Grx6, Grx7) are found to locate to the cis-Golgi preferentially [143]. Grx6 localises at both the ER and the Golgi, and Grx7 predominantly localises at the Golgi. The glutaredoxin family are thought to play roles in detoxification of oxidative damage together with GSH, GPXs, and NADPH, preventing H_2_O_2_ production [144]. The Golgi is apparently protected from damage by Grx6 and Grx7 upregulation in response to several stresses such as sodium and peroxides (as well as calcium, specifically for Grx6), but not in response to heat shock and osmotic stress [145]. Although mammalian orthologs of these genes have not yet been identified, similar enzymes may exist that determine the level of H_2_O_2_ in the Golgi.

In addition to the above-mentioned proteins, one isoform of NOS may be considered as another source of H_2_O_2_ in the Golgi. The NOS family are the enzymes that catalyse the production of NO from L-arginine and consists of three isozymes: neuronal NOS (nNOS), cytokine-inducible NOS (iNOS), and endothelial NOS (eNOS). Of these, eNOS is reported to locate to the Golgi membrane as well as the plasma membrane caveolae in endothelial cells [146,147,148]. All NOS proteins require dimerisation and several cofactors, such as nicotinamide adenine dinucleotide phosphate (NADPH), flavin adenine dinucleotide (FAD), flavin mononucleotide (FMN), calmodulin (CaM), Haeme, and tetrahydrobiopterin (BH_4_). Although NOS-derived NO underlies productions of reactive nitrogen species (RNS), a subset of free radicals, uncoupled eNOS resulting from L-arginine and/or BH_4_ deficit, lead to the production of superoxide and subsequent H_2_O_2_ (and molecular oxygen). This reaction can occur spontaneously or as SOD-catalysed dismutation [149,150].

The biological consequences of H_2_O_2_ produced in the Golgi are still elusive. Recently, a novel Golgi-targeted H_2_O_2_ specific probe termed Np-Golgi has been developed and revealed augmented H_2_O_2_ in the Golgi in hypertensive mice [151].

## 4. ROS/H_2_O_2_ and Neurodegeneration

Oxidative stress is a hallmark of several diseases, including dementia and related neurodegeneration pathologies. Among ROS, H_2_O_2_ is often considered as the central player of redox-regulated events disrupted in neurodegenerative disorders [152,153,154]. A direct link between ROS and disease is provided by the pathology associated with SOD, an enzyme that plays a role in redox homeostasis by converting superoxide anion radicals into H_2_O_2_ and oxygen. Mutations in SOD1 have been shown to cause Amyotrophic Lateral Sclerosis (ALS) [155]. SOD1 aggregation in the motor neurons seems to be caused by oxidation of two of its free cysteines in the mitochondria where oxidative stress may occur [156]. Moreover, the excess activity of SOD1 due to a third copy of its encoding gene in Down syndrome results in the accumulation of H_2_O_2_ that may reach toxic levels sufficient to promote neuronal death [157]. An extensive body of evidence implicates oxidative stress in Alzheimer’s Disease (AD). In particular, peroxiredoxin levels are altered in AD, and lipid peroxidation is found in positive correlation with brain amyloid beta (Aβ)-plaque load (the hallmark and genetic determinant of AD), suggesting a contribution of ROS to AD pathogenesis [158,159]. Furthermore, in Huntington’s disease, oxidative damage compromises low-molecular-weight antioxidant metabolism (i.e., reduced cysteine, GSH levels [160,161,162,163], and the uptake of reduced ascorbate [164,165]) and elevates the production of free radicals. Abundant evidence also suggests redox imbalance in other neurodegenerative diseases such as Parkinson’s disease [160], and in prion pathology, where a burst of ROS accompanies neurodegeneration [166]. 

Thus, the substantial association of ROS with neurodegeneration suggested the use of antioxidants as a therapeutic strategy. Scavenging ROS by administration of antioxidants has been considered as a valid therapeutic strategy for several diseases, including neurodegeneration. For instance, trolox (a water-soluble vitamin E analogue) decreases aggregation of mutant SOD1 (SOD1^G86R^), which is a causing factor of familial ALS, in vitro [167]. Ascorbic acid (vitamin C) and a natural antioxidative compound berberine-derivative can suppress Aβ aggregation and thus AD pathology [168,169]. In addition, in vivo studies have demonstrated neuroprotective effects of some antioxidants, such as rosmarinic acid, coenzyme Q10, and α-tocopherol (vitamin E), in AD, PD, and Down syndrome animal models, respectively [170,171,172,173]. 

However, despite these encouraging results using antioxidants as neuroprotectants in cell systems [174], beneficial outcomes at the organism level are inconsistent and, in large part, disappointing [175,176,177]. The reasons for those failures are not immediately clear and may have to do with their effective bioavailability and/or antioxidants’ effects on physiological roles of ROS in cell signalling, (H_2_O_2_, in particular [178,179]). Significant antioxidant beneficial effects against neurodegeneration are predominantly seen at concentrations nearing their toxicity range when redox-dependent vital signalling pathways are also likely to be perturbed [176]. Developing organelle-selective antioxidants such as 2-[2-(triphenylphosphonio)ethyl]-3,4-dihydro-2, 5,7,8-tetramethyl-2H-1-benzopyran-6-ol bromide (TPPB) and MitoQ, mitochondria-targeting antioxidants, may half-circumvent some of these limitations [180,181].

Rescuing redox homeostasis poses a challenge ahead to effective treatments against oxidative stress, which requires targeted strategies that are often confounded by the low specificity of most antioxidants in general. Favourably, metabolic antioxidants acting on specific metabolic transformations seem to have exerted alleviating effects in several animal models of neurodegenerative diseases [176]. Specific examples in cultured cell models include the apparent benefit afforded through modulating ROS-detoxifying selenoenzymes such as glutathione peroxidases, thioredoxin reductases and selenoprotein P [182,183,184]. 

Another major hurdle in developing neuroprotective antioxidants seems to be their low blood–brain barrier permeability [177]. ROS are short-lived species due to their high reactivity. Some are able to oxidise cysteine and methionine residues, the DNA base guanine is prone to oxidative damage by ROS, lipids can undergo peroxidation, and even carbohydrates are prone to oxidative degradation and depolymerisation [160]. Quenching of ROS by antioxidants to prevent oxidative damage requires fast kinetics of reduction, which are not a given for all antioxidants. The problem may be further aggravated by limited bioavailability of antioxidants at the relevant location. For instance, quenching of H_2_O_2_ by reduced glutathione in the ER becomes kinetically competitive with enzyme catalysis only at 0.23 µM of H_2_O_2_ [13]. Thus, slow kinetics of antioxidant-ROS reactions may permit oxidative damage prior to ROS quenching events.

The challenge posed is to nullify disease-relevant ROS with spatiotemporal specificity whilst leaving physiological ROS intact, which requires the drawback of antioxidant’s low-specificity to be circumvented. In targeting disease-relevant enzymatic sources of ROS, ROS-toxifiers (that convert relatively non-toxic ROS to more reactive species) or proteins that repair other oxidatively damaged proteins has been proposed as a more rational strategy to interfere with ROS than the use of antioxidants [185]. Understanding intracellular ROS–source pathways is a key to informing such strategies.

Reports establishing a connection between oxidative stress and neurodegenerative disorders are innumerous and very disparate regarding a rational and causative link between them. Whether oxidative stress elicits neurodegeneration or is a consequence of neuronal cell death triggered by a combination of genetic and epigenetic factors and environmental interactions is still a matter of debate. The table below compiles a selection of findings on these relationships (Table 1). 

### ER H_2_O_2_ and Neurodegeneration

The extent of UPR activation strongly correlates with the pathology of human patients carrying neurodegenerative diseases [231], and neurodegeneration-causing factors, such as Aβ_1–42_ oligomer and tau, can also augment UPR [232,233], suggesting that excess UPR (ER stress) synergically contributes to the development of neurodegeneration. Given that UPR is originally an adaptive signalling pathway that governs quality control of proteins in the ER, target genes of UPR should be expected to be neuroprotective. PDI in particular, is considered as a potential target UPR-gene. Indeed, PDIA3 (ERp57) protects from ischemia-induced brain damage [234]. Further, upregulation of PDI expression in the central nervous system (CNS) seems to be a common feature observed in the patients and animal model of several neurodegenerative diseases such as AD, PD, ALS, and HD [235,236,237,238,239]. Interestingly, reversible/irreversible inhibitors of PDI show neuroprotective effects in vitro [240,241]. In addition, inhibitions of PDI by CCF642 or LOC14 appear neuroprotective in a mouse model of Experimental autoimmune encephalomyelitis (EAE) and HD [242,243]. ERO1 inhibitor EN460 also antagonises neurotoxicity [244]. Seemingly paradoxical observations that both inhibitors of these proteins and their upregulation show neuroprotective effects [169,245,246] can be reconciled under the assumption that their activity is crucial, but their by-product-ROS is hazardous. Consistently, small molecule antioxidants relieve not only oxidative load but also ER stress [247], probably at least in part by removing PDI and ERO1-derived ROS.

Further, ER-antioxidative enzymes benefit neuronal health: PRDX4 can protect from Aβ oligomer- or glutamate-mediated cell death presumably by suppressing ER stress [38,248]. While GPX7 deficient mice show an accelerated, aging-related neurodegeneration, accompanied by elevated ROS, in spinal motor neuron [245]. A brain transcriptome analysis reveals that this gene is significantly upregulated in response to neurotoxin MPTP [246,249].

**Figure 2:**
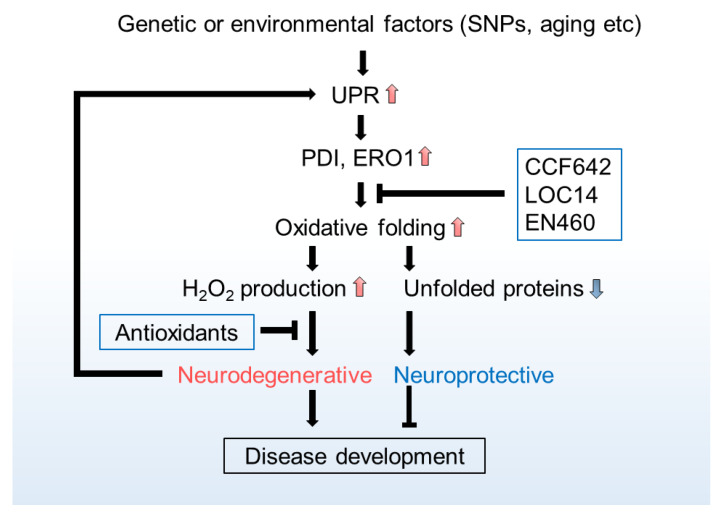
Schema of unfolded proteins response (UPR)-induced neurodegeneration and potential therapeutic strategies. UPR activated by a variety of factors, such as single nucleotide polymorphisms (SNPs) as genetic factors and aging or metabolic dysfunction as environmental factors, augments the expressions of target genes such as PDI and ERO1, and enhances the oxidative folding capacity of the ER. This can reduce the accumulation of unfolded proteins and results in neuroprotection, whereas excess H_2_O_2_, which is concomitantly produced by this process, exacerbates neurodegeneration as an oxidative stress agent. Inhibitors against PDI or ERO1 (such as CCF642, LOC14, and EN460) and antioxidants are considered promising in mitigating the development of neurodegenerative pathology by reducing ER-derived H_2_O_2_.

Together these observations suggest that if excess ER-ROS can be eliminated from the cell, the beneficial outcomes of UPR should be expected to antagonise the neuropathophysiology more effectively (Figure 2).

## 5. Conclusions and Perspectives

In this review, we have outlined the sources and known functionalities of ROS from the ER and other organelles. Organelles harbour pathways that produce ROS as a by-product of redox reactions and/or to be utilised as a signalling factor. Uniquely, the ER utilises a ROS, H_2_O_2_ to directly fuel is anabolic activity—the oxidative protein folding. This organelle satisfies its own demand of ROS by internally producing abundant H_2_O_2_, on a scale that may well exceed that of any other organelle. As the ER constitutes a major source of ROS in the cells, the organelle’s homeostasis often correlates with cellular redox, explaining the simultaneous presence of ER stress and oxidative stress in the process of ageing and diseases.

It is plausible that antagonising ROS with antioxidants may have utility in alleviating the oxidative damage in certain disease states. However, the use of antioxidants as a therapeutic strategy is still controversial due to the uncertainty of their usefulness in humans. This may be because the comprehensive scavenging of systemic ROS not only relieves oxidative stress but also deprives the physiological advantages of ROS, which drive biological reactions by physiological oxidation of target molecules. Although distinguishing ROS benefit and toxicity in vivo is a significant challenge, this dilemma must be resolved to some extent by source-selective suppression of excessive ROS. As mentioned in this review, some molecules have indeed been developed such as TPPB and mitoQ (mitochondria-targeting antioxidants) and CCF642, LOC14, and EN460 (PDI and ERO1 inhibitor, respectively) to decrease local ROS productions. Therefore, controlling spatial ROS productions will be a future focus for providing antioxidants a utility as therapeutics. Finally, visualisation of ROS in live-cells has become clearer by the development of organelle-targeted genetically encoded redox sensors, such as roGFP and HyPer, as well as chemical probes. Most of these probes can detect ROS reversibly, allowing the monitoring of homeostatic ROS production and consumption. Indeed, these techniques also capture the redox state of tissues in vivo [250,251]. In addition to these tools, further development of biosensor implementations of image acquisition technology such as two-photon microscopy, super-resolution microscopy, and single-molecule analysis will likely enable mapping of physiological ROS with higher spatiotemporal resolution. The elucidation of local ROS functionality will be expected to provide new clues to aid our understanding of the pathophysiology not only of neurodegenerative diseases, but also ageing, cancer, and other related diseases.

## Figures and Tables

**Table 1:** Potential relations between oxidative stress and neurodegenerative disorders.

Neurodegenerative Disorder	Possible Source of Oxidative Stress	Cell Component Affected	Reference
Parkinson disease	Not determined	Oxidized DJ-1 ^1^ protein	[186]
	H_2_O_2_/rotenone/6-hydroxydopamine	Parkin	[187]
	Ca^2+^ influx through L-type channels/α-synuclein intracellular inclusions	Mitochondrial oxidant stress	[188,189]
	NADPH oxidase	α-synuclein	[190]
	NOS ^2^	S-nitrosylation of PDI ^3^	[191]
	Not determined	Depletion of GSH	[192,193,194]
	Intrastriatal dopamine injections	Protein-bound cysteinyl catechols/Loss of tyrosine hydroxylase	[195]
	Loss of dopamine homeostasis	Inhibition of NADH oxidase/mitochondrial dysfunction	[196]
	Formation of the hexanoyl dopamine adduct	Mitochondrial abnormality/apoptosis	[197]
	α-synuclein oligomer-induced ROS production	Depletion of GSH	[198]
	α-synuclein oligomers	Mitochondrial dysfunction	[199]
	Not determined	Mitochondrial respiratory-chain Complex I	[200]
	Iron deposition in deep brain nuclei	Fibrillation of α-synuclein/dopaminergenic neurodegeneration	[201,202]
	Inflammogen lipopolysaccharide/ NOS ^2^ and NADPH oxidase	Aggregated nitrated α-synuclein/upregulation of inflammatory markers	[203]
	Extracellular aggregated α-synuclein	Activation of NADPH oxidase/microglial activation	[204]
	Activation of NOS ^2^	Decrease in parkin sulfhydration/increase in parkin nitrosylation	[205]
	Nitric oxide	S-nitrosylation of Prx2 ^4^	[206]
Alzheimer’s disease	Not determined	Oxidized DJ-1 ^1^ protein	[186]
	Aβ plaques	Neurites	[207]
	Aβ plaques	Reduced GSH levels/Decreased SOD activity	[208]
	Aβ plaques	Mitochondrial protein import channels	[209]
	Not determined	Nuclear and mitochondrial DNA damage	[210]
	Increase in BACE1 ^5^ activity	Lipid peroxidation	[211]
	Not determined	JNK/SAPK ^6^ dysregulation	[212]
	Aβ oligomers	Inhibition of EAAT3 ^7^-mediated cysteine uptake/decreased GSH levels/decreased DNA methylation	[213,214]
	Inhibition of GSH synthesis	Increased levels of tau phosphorylated	[215]
	Age-dependent	Mitochondrial DNA deletion	[216]
	Not determined	Loss of REST ^8^	[217]
	NOS ^2^	S-nitrosylation of PDI ^3^	[191]
	Aβ(1-42) oligomers	Activation of NMDARs ^9^/increase in Nitric oxide	[218]
Huntington’s disease	Age-dependent	Mitochondrial DNA damage	[219]
	Not determined	Cytosolic SOD ^10^ activity/nuclear DNA/oxidative phosphorylation enzymes	[220]
	Not determined	Cu/Zn-SOD ^10^ activity/glutathione peroxidase activity/mitochondrial DNA	[221]
	Not determined	Reduced GSH levels/plasma lipid peroxidation	[222]
	Mitochondrial Ca^2+^ signalling and superoxide generation	Mitochondrial DNA damage	[223]
	Depletion of cystathione γ-lyase ^11^	Low levels of GSH/protein carbonylation/protein nitration/lipid peroxidation	[161]
	Impaired cysteine biosynthesis and transport	Failure of activating transcriptional factor 4 (ATF4) induction	[163]
	Uptake of vitamin C	Impaired trafficking of vitamin C transporters/impaired glucose transporter GLUT3 to the membrane	[164,165]
	Iron metabolism	Not determined	[224]
	Age-dependent	Oxidative DNA damage (CAG trinucleotide expansion)	[225]
	Reduced activity of Nrf2 ^12^	Impaired mitochondrial dynamics/decreased mitochondrial fusion	[226]
	Impaired function of PGC1α ^13^	Mitochondrial dysfunction	[227]
	Not determined	Reduced levels of GSH	[228]
Amyotrophic lateral sclerosis	Mutations in Cu/Zn SOD ^10^ (familiar cases)	Increased cytosolic and mitochondrial ROS/impaired mitochondrial respiration	[229,230]

^1^ Ubiquitously expressed protein of the DJ-1/ThiJ/PfpI superfamily whose biochemical function is unknown; ^2^ Nitric oxide synthase; ^3^ Protein disulphide isomerase; ^4^ Peroxiredoxin-2; ^5^ β-secretase, β-site amyloid precursor protein cleaving enzyme 1; ^6^ c-Jun N-terminal kinase/Stress activated protein kinase; ^7^ Excitatory amino acid transporter 3; ^8^ Repressor element 1-silencing transcription factor, represses genes that promote cell death and induces expression of stress response genes; ^9^ N-methyl-D-aspartate-type glutamate receptor; ^10^ SOD—superoxide dismutase; ^11^ Biosynthetic enzyme for cysteine; ^12^ Erythroid 2-related factor 2, a master transcriptional regulator of the cellular antioxidant stress response; ^13^ Peroxisome proliferator-activated receptor γ coactivator 1α, a master transcriptional co-regulator of mitochondrial biogenesis and metabolism and antioxidant defences.

## Glossary

Superoxide (O_2^−^_)	A radical ROS produced by the one-electron reduction of molecular oxygen. It is converted to hydrogen peroxide and molecular oxygen by spontaneous and/or SOD-catalysing dismutation.
Hydrogen peroxide (H_2_O_2_)	A non-radical, relatively stable and functional ROS, hazardous in excess.
Hydroxyl radical (·OH)	Highly reactive and short-lived radical. Its production is driven by Fenton reaction, between ferrous ion (Fe^2+^) and hydrogen peroxide.
Redox potential	Expressed in millivolts (mV), a measure of redox state that reflects the tendency of a chemical to receive/donate electrons, and concentrations of its reduced and oxidised species, given by the Nernst equation
Superoxide dismutase (SOD)	An antioxidant enzyme catalysing dismutation of superoxide to hydrogen peroxide and molecular oxygen. SOD family consists of Cu/Zn-SOD (SOD1), Mn-SOD (SOD2), and EC-SOD (SOD3). Mutations in the SOD1 gene affect approximately 20% of familial amyotrophic lateral sclerosis (ALS).
Peroxiredoxin (PRDX)	An antioxidant enzyme scavenging peroxides including H_2_O_2_. PRDX family consists of six isoforms (PRDX1-6). PRDX4 is localised in the ER.
Glutathione peroxidase (GPX)	An antioxidant enzyme scavenging H_2_O_2_ as well as lipid peroxides by utilising reduced glutathione. GPX family consists of eight isoforms (GPX1-8). GPX1-4 and GPX6 contain an active site selenocysteine (selenoproteins). GPX7 and GPX8 are localised in the ER. GSH peroxidase activity of GPX7 and GPX8 is low.
Glutathione	An abundant antioxidant tripeptide consisting of alanine–cysteine–glutamate (with cysteine and glutamate linked by gamma–glutamyl bond). Reduced glutathione (GSH) drives GPXs reactions and as a result GSH is converted to the oxidised form (GSSG), in which two molecules are bound by disulfide bond. GSSG is recycled by reducing by glutathione reductase (GR).
Catalase	An antioxidant enzyme that scavenges H_2_O_2_. The iron-bound heme group in the active site catalyses the conversion of H_2_O_2_ to water (H_2_O) and molecular oxygen (O_2_). It mainly localises in the peroxisomes.
Protein disulfide isomerase (PDI)	An ER chaperone possessing oxidoreductase activity via CXXC catalytic motif-containing thioredoxin domains. Catalyses protein disulphide bond formation and isomerisation during folding of ER client proteins.
ER oxidoreductin 1 (ERO1)	A sulfhydryl oxidase that supplies oxidising equivalents to recycle PDI, in the process ERO1 produces H_2_O_2_ by reducing O_2_ with its active site (CXXCXXC motif/flavin adenine dinucleotide (FAD) prosthetic group).
NADPH (nicotinamide adenine dinucleotide phosphate) oxidase (NOX)	An enzyme catalysing the production of a superoxide by transferring one electron to oxygen from NADPH. The NOX family consists of NOX1~NOX5, Dual oxidase 1 (DUOX1) and DUOX2. Among NOXs, NOX4 preferentially produces H_2_O_2_ rather than superoxide.
